# The Past, Present, and Future of Metal Halide Perovskite Light‐Emitting Diodes

**DOI:** 10.1002/smsc.202000072

**Published:** 2021-05-07

**Authors:** Michael Worku, Azza Ben-Akacha, Tunde Blessed Shonde, He Liu, Biwu Ma

**Affiliations:** ^1^ Materials Science and Engineering Program Florida State University Tallahassee FL 32306 USA; ^2^ Department of Chemistry and Biochemistry Florida State University Tallahassee FL 32306 USA

**Keywords:** charge transport, efficiency roll-off, lead-free perovskite materials, light-emitting diodes, metal halide perovskites, stability

## Abstract

Metal halide perovskites (MHPs) have emerged as new‐generation highly efficient narrow‐band luminescent materials with applications in various optoelectronic devices, including photovoltaics (PVs), light‐emitting diodes (LEDs), lasers, and scintillators. Since the demonstration of efficient room‐temperature electroluminescence from MHPs in 2014, remarkable progress has been achieved in the development and study of light‐emitting MHP materials and devices. While the device efficiencies of MHP LEDs (PeLEDs) have significantly improved over a short period of time, their overall performance has not reached the levels of mature technologies yet, such as organic LEDs (OLEDs) and quantum dot LEDs (QDLEDs), to enable practical applications. Many issues and challenges, including low operational stability, lack of efficient blue PeLEDs, and toxicity of MHPs, remain to be addressed. Herein, some of the most exciting progress achieved in the development of efficient and stable PeLEDs during the last few years are introduced, the main issues and challenges in the field are discussed, and the prospects on addressing these issues and challenges are provided. With continuous effort, the potential of PeLEDs to become a commercially available LED technology for display and lighting applications in the future looks optimistic.

## Graphical abstract

Metal halide perovskite light‐emitting diodes are promising devices for next‐generation display applications. Although their rapid development is exciting, they have to overcome several challenges to be competitive with more mature technologies. Herein, some of the remaining challenges are outlined and routes that can be explored to address these challenges are proposed.

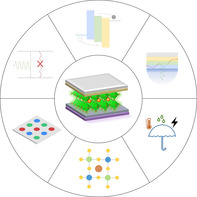

## 1 Introduction

**Figure 1:**
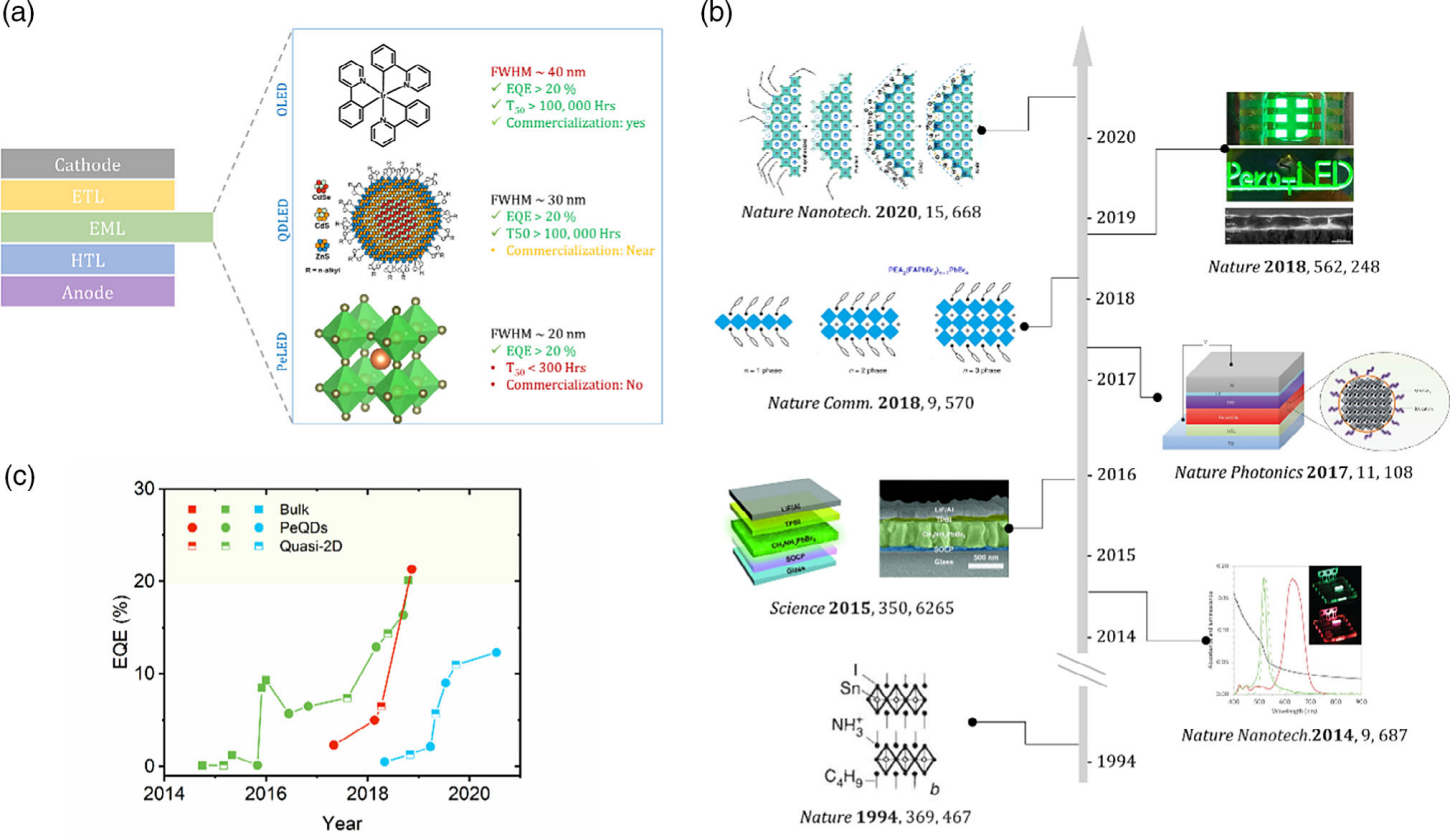
a) Schematic showing the typical device structure of thin‐film LEDs and EML for OLEDs, QDLEDs, PeLEDs, b) representative works in PeLEDs over the years, c) trends of PeLED EQEs for red, green, and blue emissions. a) QDLED: Reproduced with permission.^[^ ^156^ ^]^ Copyright 2017, American Chemical Society. b) From bottom to top (in the order of year): Reproduced with permission.^[^ ^154^ ^]^ Copyright 1994, Springer Nature. Reproduced with permission.^[^ ^11^ ^]^ Copyright 2014, Springer Nature. Reproduced with permission.^[^ ^12^ ^]^ Copyright 2015, American Association for the Advancement of Science. Reproduced with permission.^[^ ^62^ ^]^ Copyright 2017, Springer Nature. Reproduced under the terms of the CC‐BY 4.0 license.^[^ ^155^ ^]^ Copyright 2018, The Authors, published by Springer Nature. Reproduced with permission.^[^ ^13^ ^]^ Copyright 2018, Springer Nature. Reproduced with permission.^[^ ^18^ ^]^ Copyright 2020, Springer Nature.

Electrically driven thin‐film light‐emitting diodes (LEDs), where light‐emitting films are sandwiched between charge transporting layers (CTLs) and electrodes (**Figure** **1**a), have been sought after for decades as alternatives to conventional compound semiconductor‐based LEDs. These LEDs are especially attractive for display applications requiring high efficiency, tunable and vivid colors, as well as cost‐effective mass production. The tremendous success in the development and application of organic LEDs (OLEDs) technology has made it one of the top technological innovations since 1980 that has dramatically affected everyday life. Although reports of electroluminescence from organic materials date back to the 1960s, it was not until Tang's breakthrough work on vapor‐deposited bilayer OLEDs that low‐voltage operable organic thin‐film LEDs were realized.^[^ ^1^ ^]^ This discovery catapulted the field of OLEDs to the invention of fluorescent polymer LEDs (PLEDs),^[^ ^2^ ^]^ followed by phosphorescent OLEDs (PHOLEDs),^[^ ^3^ ^]^ and more recently thermally activated delayed fluorescence (TADF) OLEDs.^[^ ^4^ ^]^ Although OLEDs afford high brightness and high‐efficiency displays with long operational lifetimes, the demand of higher color vividity for displays requires LEDs with narrower emission widths than those of OLEDs. Moreover, the use of rare noble metals for display applications is unsustainable and thin‐film LEDs based on Earth‐abundant materials are desired.

Following suit, thin films based on metal chalcogenide quantum dots (QDs) have also been shown to produce efficient electroluminescence, although commercial display products are not available yet. This class of materials are especially interesting for display applications for their higher color purity as compared to organic emitters, as well as their higher thermal and chemical stability. With two decades of research and development efforts, QD‐based LEDs (QDLEDs) with emissions across the entire visible spectrum have been reported to exhibit internal quantum efficiencies (IQEs) close to the theoretical maxima^[^ ^5^, ^6^, ^7^ ^]^ and long operational lifetimes meeting the standards for commercial products. However, most efficient and stable QDLEDs are based on QDs containing cadmium, a regulated toxic metal in the EU and US. Thus, a significant amount of efforts have been focused on finding nontoxic QDs for QDLEDs with high device efficiencies and operational stability. Recently, researchers at Samsung demonstrated highly efficient red QDLEDs based on InP core–shell QDs with external quantum efficiencies (EQEs) of >20% and a very impressive lifetime of 1 000 000 h at 100 cd m^−2^.^[^ ^8^ ^]^ The same group also demonstrated blue QDLEDs with EQEs of ≈20% and a long lifetime of over 15 000 h at 100 cd m^−2^ using a ZnSe‐based core–shell QD system.^[^ ^9^ ^]^ It is well expected that commercial display products based on QDLEDs will soon be available in the market.

The successes of OLEDs and QDLEDs have not stopped researchers from exploring new generation thin‐film LEDs. Metal halide perovskites (MHPs) have received great attention as new‐generation light emitters for thin‐film LEDs, considering their tunable narrow emissions, excellent charge transport properties, defect tolerance, and facile solution processability. The perovskite crystal structure was first discovered in CaTiO_3_ in 1839 by Gustav Rose and was named after the Russian geologist Lev Perovski. Typical MHPs have a general chemical formula of ABX_3_, where A represents the monovalent cation, such as methylammonium (CH_3_NH_3_^+^), Cs^+^, and formamidinium (CH(NH_2_)_2_^+^), B a divalent metal ion, such as Pb^2+^ and Sn^2+^, and X a halide anion (Cl^−^, Br^−^, I^−^, or their mixtures). Besides ABX_3_ MHPs with a 3D structure, MHPs have been used to describe organic metal halide hybrids with 2D and quasi‐2D structures, or so‐called layered‐2D and quasi‐2D MHPs, in which the metal halide layers are separated by organic cation layers to exhibit quantum confinement effects. Although early work on using MHPs for thin‐film LEDs dates back to the 1990s by Mitzi and coworkers,^[^ ^10^ ^]^ room‐temperature electroluminescence from PeLEDs was not demonstrated until 2014 by Tan et al. with EQEs of less than 1%.^[^ ^11^ ^]^ A major leap was recorded when Lee and coworkers used a nanocrystal (NCs) pinning technique to achieve in situ formation of MAPbBr_3_ nanograins that increased the exciton binding energy and led to efficient green PeLEDs with EQEs of more than 8%.^[^ ^12^ ^]^ A timeline showing some of the representative advances in PeLED development is shown in Figure 1b. To date, highly efficient green, red, and near‐infrared (NIR) PeLEDs with IQEs approaching the theoretical maxima have been demonstrated.^[^ ^13^, ^14^, ^15^, ^16^ ^]^ Remarkable progress has also been achieved in efficient blue PeLEDs within a short period of time,^[^ ^17^, ^18^ ^]^ although their performance is not yet comparable with those of green, red, and NIR ones. The trends in PeLED efficiency over the years are shown in Figure 1c. Unlike the device efficiency of PeLEDs reaching to the levels of OLEDs and QDLEDs, the device stability of PeLEDs is far behind, with the longest reported half‐lifetime (*T*_50_) of a few hundred hours compared with >100 000 h for OLEDs and QDLEDs. Moreover, several issues and challenges, including toxicity, environmental, and spectral stability, as well as the development of processing and patterning techniques compatible with mass manufacturing must be addressed before possible commercialization of PeLEDs. In this Perspective, we highlight the recent progress in PeLEDs and discuss what we can learn from OLEDs and QDLEDs to address the issues and challenges in PeLEDs to advance the technology toward commercialization.

## 2 The Road to Commercialization

The main roadblocks to the commercialization of PeLEDs can be summarized as those related to device efficiency, device stability, toxicity, and mass production. In this section, we highlight the notable strategies explored to address the aforementioned issues.

### 2.1 Device Efficiency

Device efficiency of PeLEDs is a function of the radiative efficiency of the emitting layer (EML), the charge balance in the EML, and the fraction of outcoupled light from the device stack in the front direction. To obtain highly efficient PeLEDs, each of these factors must be optimized accordingly. Here, we highlight notable achievements in the development of color‐tunable MHP EMLs and CTLs, as well as strategies for the improvement of efficiency roll‐off and light extraction, and outline the remaining issues and challenges.

#### 2.1.1 Emitting Layer

##### Bandgap Control and Emission Color Tuning

The narrow emission width of MHPs (≈20 nm), compared with those of typical QDs (≈30 nm) and organic emitters (>40 nm), makes them especially attractive for high‐color purity next‐generation display applications. The versatility of MHPs in energy bandgap and emission color tuning also makes them very promising to meet the requirements set by BT Recommendation 2020.^[^ ^19^ ^]^ The ability of readily obtaining green and NIR emissions from pure bulk MHPs, such as MAPbBr_3_ and MAPbI_3_, has led to the rapid development of highly efficient green and NIR PeLEDs with EQEs of >20%.^[^ ^13^, ^14^, ^15^, ^20^ ^]^ To obtain other colors in the visible spectrum, bandgap control of MHPs can be realized in multiple ways through compositional, morphological, and dimensional control.

**Figure 2:**
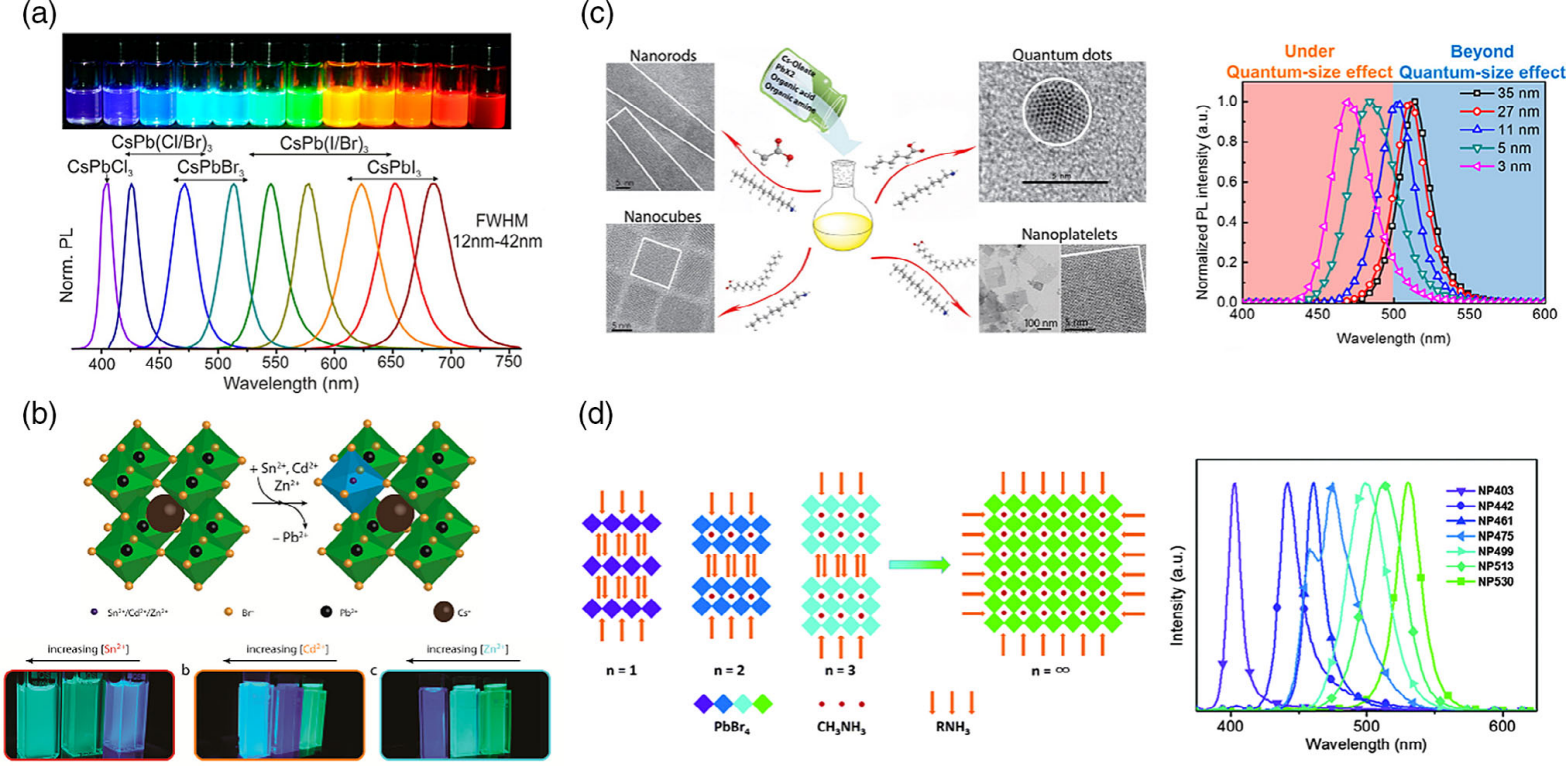
Emission tuning strategies: a) compositional engineering through halide mixing and exchange and b) B‐site substitution, c) Left: quantum confinement through the formation of QDs, nanorods, and nanoplatelets. Right: emission tuning through quantum confinement. d) Formation of layered‐2D and quasi‐2d structures. a) Reproduced with permission.^[^ ^26^ ^]^ Copyright 2015, American Chemical Society. b) Reproduced with permission.^[^ ^157^ ^]^ Copyright 2017, American Chemical Society. c) Left: Reproduced with permission.^[^ ^158^ ^]^ Copyright 2016, American Chemical Society. Right: Reproduced with permission.^[^ ^159^ ^]^ Copyright 2017, American Chemical Society. d) Reproduced with permission.^[^ ^31^ ^]^ Copyright 2016, The Royal Society of Chemistry.

Compositional control, especially through halide mixing, is a simple approach that has been used to obtain blue^[^ ^21^ ^]^ and red PeLEDs (**Figure** **2**a).^[^ ^16^ ^]^ For instance, Kido and colleagues used a modified hot injection method to synthesize mixed halide CsPb(Br/I)_3_ NCs with high photoluminescence quantum efficiencies (PLQEs) and fabricate pure red PeLEDs (peak emission at 653 nm) with EQEs of >20%.^[^ ^16^ ^]^ However, light‐ and electrical field‐induced ion migration and phase segregation lead to spectral instability that is undesirable for display applications. Substitution in the B^2+^ site has also been shown as an effective approach for bandgap control and emission color tuning, for instance, partially replacing Pb^2+^ with Sn^2+^, Cd^2+^ and Zn^2+^ (Figure 2b),^[^ ^22^ ^]^ as well as heterovalent substitution of Pb^2+^ with Al^3+^ in MHP NCs.^[^ ^23^ ^]^ Rogach and coworkers have demonstrated red PeLEDs with EQEs of >15%, by introducing varying amounts of Zn^2+^ in CsPbI_3_ NCs.^[^ ^24^ ^]^ While this strategy seems to be working well, the high formation energy of B^2+^‐alloyed MHPs makes thin‐film formation nontrivial.^[^ ^25^ ^]^

Synthesizing quantum‐confined MHP NCs with controlled topologies and sizes represents another effective way of bandgap control and emission color tuning.^[^ ^26^ ^]^ Highly luminescent MHP 2D nanoplatelets, 1D nanowires, and 0D QDs with tunable emissions, as a result of quantum size effects, have been well reported (Figure 2c).^[^ ^27^, ^28^ ^]^ For instance, the emission of pure bromide‐based MHP nanoplatelets can be blue shifted by as much as 0.6 eV via size control to yield blue emission. Blue PeLEDs based on CsPbBr_3_ nanoplatelets with thickness of less than 4 nm have been demonstrated by several groups, although the device performance is relatively low.^[^ ^29^, ^30^ ^]^ Similarly, small‐sized CsPbBr_3_ QDs with strong quantum confinement can also exhibit blue emissions and have been used in blue PeLEDs with record EQEs.^[^ ^18^ ^]^

Another strategy of bandgap control is the formation of MHPs with reduced dimensionalities at the molecular level, particularly layered‐2D and quasi‐2D MHPs that have been shown to exhibit highly efficient tunable emissions with excellent film formation (Figure 2d).^[^ ^31^, ^32^ ^]^ Because of the large charge transport barrier and strong electron−phonon interaction in layered‐2D MHPs,^[^ ^33^, ^34^ ^]^ quasi‐2D MHPs have been more popular for electroluminescent devices. However, solution processing of quasi‐2D MHPs often leads to the formation of multiple phases with varying thicknesses and bandgaps. Because of the fast charge/energy funneling in these systems,^[^ ^35^ ^]^ emission is ascribed to the phases with the lowest bandgaps, which can be 3D MHP phases, limiting their emission color tunability. These mixed phases in quasi‐2D MHPs also result in emissions much broader than those of bulk and nanocrystalline 3D MHPs. Recently, several groups have shown the use of additives for phase control of solution‐processed quasi‐2D MHPs to increase the emission color purity and tunability.^[^ ^36^ ^]^ For instance, our group used diammonium salts to modulate the formation of multiple quantum well phases to achieve tunable emissions in the blue region, in which the interaction of diamine with undercoordinated Pb^2+^ ions restricts the growth of thick phases. Developing new strategies to obtain pure quasi‐2D MHPs via solution processing would be of great interest in this regard.

##### Formation of EML

**Figure 3:**
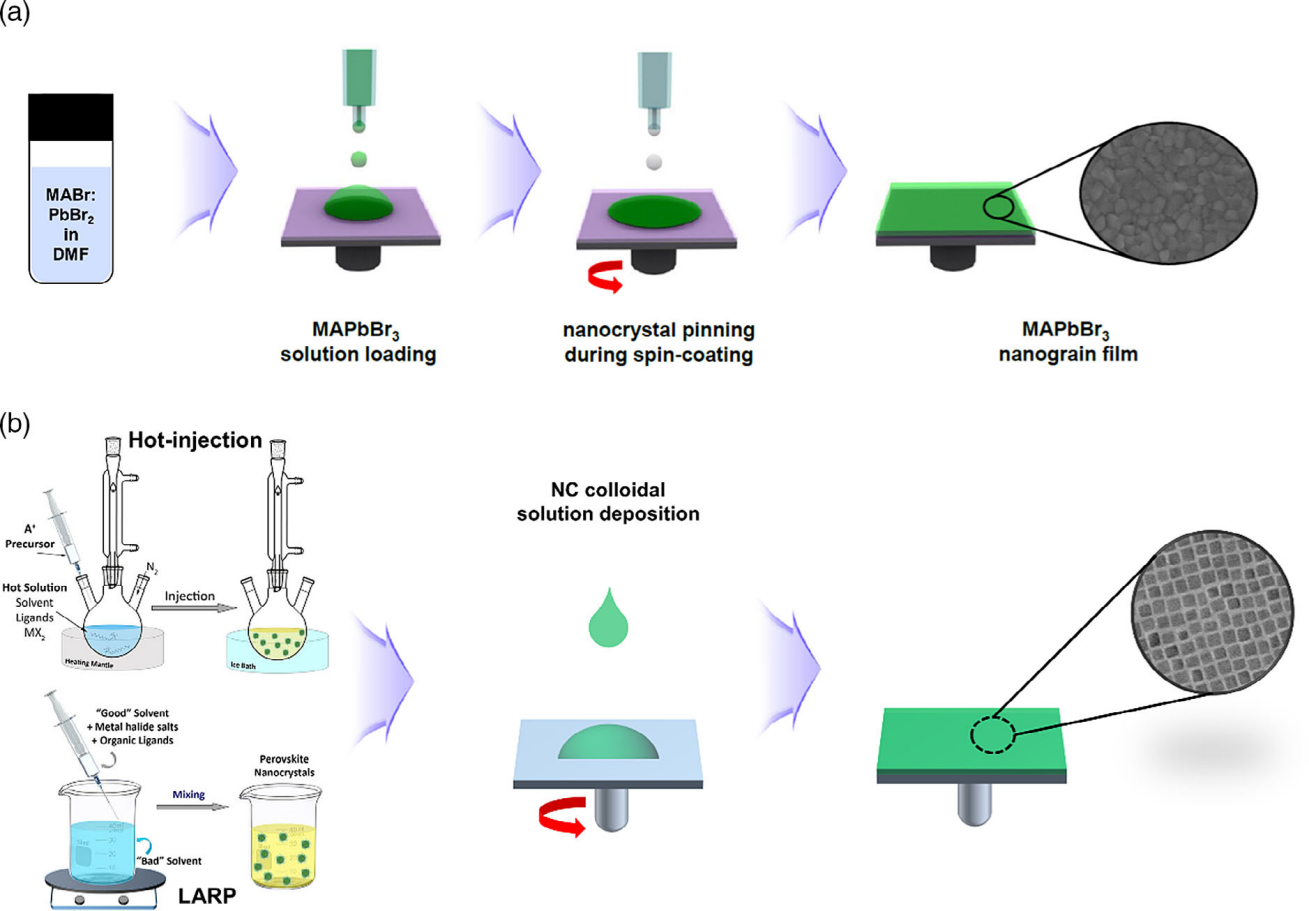
EML formation: a) in situ MHP NCs formation, b) ex situ MHP NCs synthesis through hot injection or ligand‐assisted reprecipitation (LARP) methods followed by thin‐film deposition. a) Reproduced with permission.^[^ ^12^ ^]^ Copyright 2015, American Association for the Advancement of Science. b) Reproduced with permission.^[^ ^44^ ^]^ Copyright 2019, American Chemical Society.

Besides emission color tuning, much work has been focusing on obtaining defect‐free MHP EMLs with high PLQEs and charge transport properties. To date, preparation of MHP EMLs for highly efficient PeLEDs has relied on two major approaches, one based on solution processing of MHP precursors with in situ formation of crystalline MHPs and the other involving solution processing of presynthesized MHP NCs (**Figure** **3**). For MHP EMLs prepared via in situ formation, blending MHP precursors with appropriate molecular/polymeric additives to form surface‐passivated crystalline MHP domains has achieved great success.^[^ ^15^, ^37^, ^38^, ^39^, ^40^, ^41^, ^42^ ^]^ In these MHP EMLs, extrinsic defects, such as grain boundaries and surface defect states, and intrinsic point defects, such as vacancies, antisites, and interstitials, could be significantly reduced, resulting in high PLQEs. Our group developed high‐performance MHP EMLs via solution processing of MAPbBr_3_ MHP precursors together with organic phosphonium ligands. In these organic−MHP composite thin films, the in situ‐formed MHP NCs are uniformly embedded in the organic matrix and surface passivated to exhibit significantly enhanced luminescence and stability, as compared with neat MHP thin films.^[^ ^43^ ^]^ Huang and coworkers reported one of the most efficient PeLEDs to date with a peak EQE of 20.7%, in which the MHP EMLs with submicrometre‐scale structures were formed by introducing amino acid additives into the MHP precursor solutions. The additives not only control the crystal growth kinetics, but also effectively passivate MHP surface defects.^[^ ^14^ ^]^ For MHP EMLs prepared by solution processing of presynthesized colloidal MHP NCs, high PLQEs could be easily achieved but obtaining smooth film morphology or good charge transport properties is more challenging. To achieve high PLQEs for MHP NCs with high surface area, a large amount of organic ligands are often needed to ensure low surface trap density and colloidal stability, which in turn result in poor charge transport properties.^[^ ^44^ ^]^ To address this issue, inorganic passivation, where the long insulating organic chains are replaced by inorganic species, has been developed as a highly promising approach to realize both high PLQEs and charge transport properties.^[^ ^45^, ^46^ ^]^ Recently, Sargent and coworkers demonstrated blue PeLEDs (peak emission at 479 nm) with a record EQE of 12.3% using a bipolar shelling strategy that achieves both surface trap passivation and good charge transport.^[^ ^18^ ^]^ Besides optimal optical, morphological, and electronic properties, obtaining EMLs with high environmental and thermal stability is another area that requires research focus.

#### 2.1.2 Charge Transport Layers

**Figure 4:**
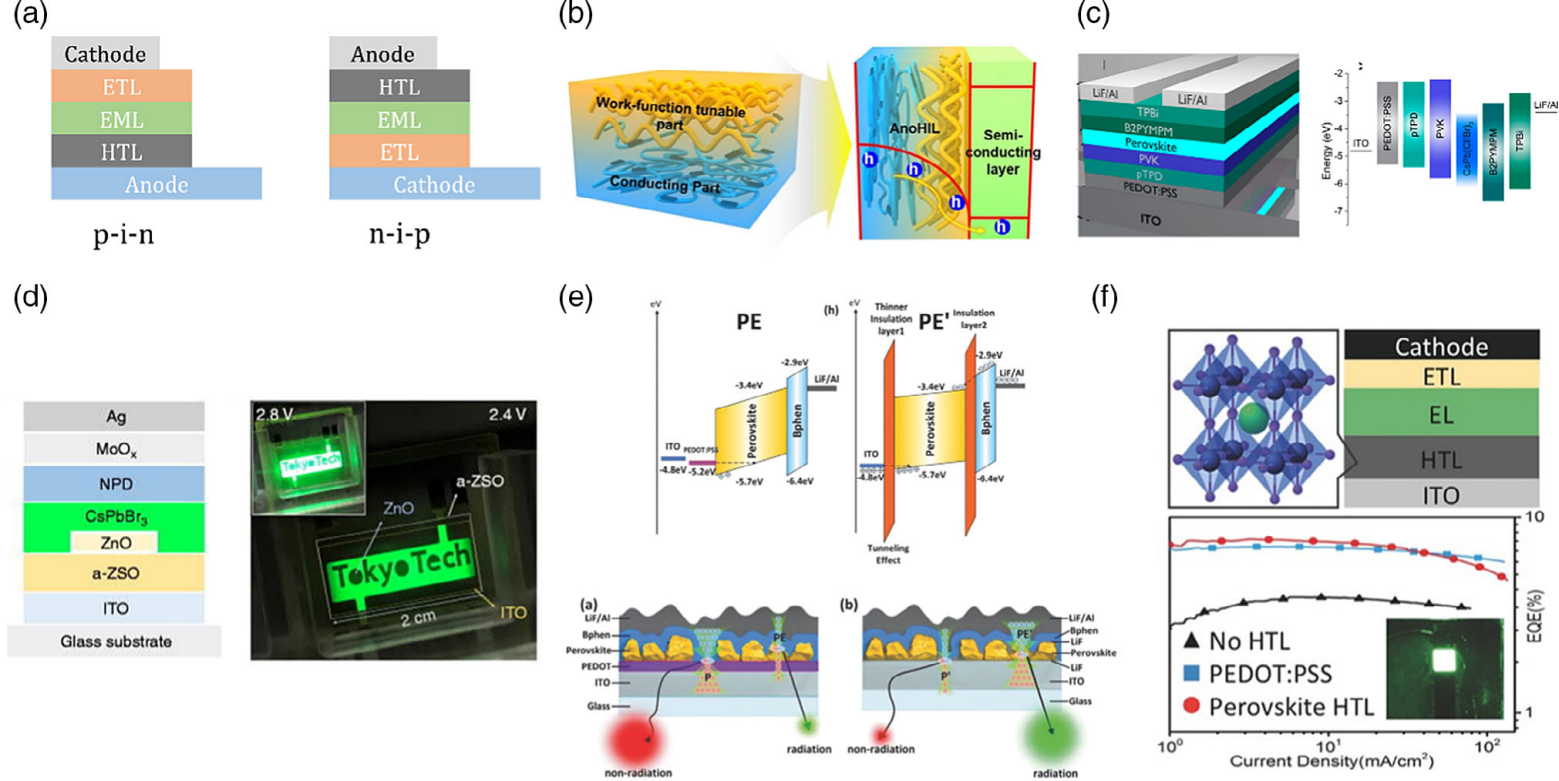
a) Standard (left) and inverted (right) device architectures. b) The schematics of spin‐coated PEDOT:PSS:PFI, implying a combination of anode and hole injection layer that shows self‐organization and hole injection to an overlying semiconducting layer. c) Schematic of blue PeLED using small organic/polymeric CTLs. d) Left: device structure of large PeLED where a 20 nm‐thick ZnO layer was deposited on the Zn−Si−O ETL; right: photograph of large‐area PeLED under operation, e) top: device structures for standard and IPI PeLEDs; bottom: charge leakage blocking mechanism in standard and IPI PeLEDs, f) top: schematic device structure of an OLED with a neat MAPbCl_3_ thin‐film HTL; bottom: comparison of PHOLED EQEs for the different HTLs. b) Reproduced under the terms of the CC‐BY 4.0 license.^[^ ^160^ ^]^ Copyright 2017, The Authors, published by Springer Nature. c) Reproduced with permission.^[^ ^161^ ^]^ Copyright 2019, American Chemical Society. d) Reproduced with permission.^[^ ^57^ ^]^ Copyright 2019, AIP Publishing. e) Reproduced with permission.^[^ ^76^ ^]^ Copyright 2018, Wiley‐VCH. f) Reproduced with permission.^[^ ^78^ ^]^ Copyright 2016, Wiley‐VCH.

The basic device structures of PeLEDs are the same as those of OLEDs and QDLEDs, which could be classified as standard (p−i−n) or inverted (n−i−p) devices, depending on how different functional layers are stacked (**Figure** **4**a). To achieve efficient LEDs, high‐performance CTLs (hole transport layer [HTL] and electron transport layer [ETL]) are as important as the EML. Ideally, CTLs are required to achieve balanced and efficient charge injections into the EML, as well as confine excitons within the EML without interfacial emission quenching. To date, most CTLs used in PeLEDs are inherited from those used in OLEDs and QDLEDs. While the adoption of existing device structures and CTLs has contributed greatly to the fast development of efficient PeLEDs, developing new charge transport materials suitable for MHPs is needed to further advance the field.

The most common device structure in PeLEDs used poly(3,4‐ethylenedioxythiophene) polystyrene sulfonate (PEDOT:PSS) as HTL, 2,2′,2″‐(1,3,5‐benzinetriyl)‐tris(1‐phenyl‐1‐H‐benzimidazole) (TPBi) as ETL, and LiF as electron‐injection buffer layer, with indium tin oxide (ITO) and aluminum as anode and cathode, respectively. Based on this standard structure, several optimizations and modifications have been made over the years to improve the device performance of PeLEDs, by adjusting the interfaces and band alignments between EMLs and CTLs to maximize exciton formation with minimal interfacial emission quenching. For instance, the acidic nature of PEDOT:PSS can etch ITO, leading to emission quenching and device degradation by indium‐ion diffusion.^[^ ^47^, ^48^ ^]^ Moreover, the band alignment of PEDOT:PSS with MHP valence band maximum (VBM) is less than ideal, resulting in a large hole injection barrier. The PEDOT:PSS surface is also not conducive to MHP crystal growth, which leads to grains with high defect density.^[^ ^48^, ^49^ ^]^ To address all these issues related to PEDOT:PSS, a great deal of research effort has been on its modification by introducing additives and interlayers, as well as searching for alternative materials. The use of perfluorinated ionomer (PFI)^[^ ^50^ ^]^ (Figure 4b), poly(styrenesulfonate)‐grafted polyaniline (PSS‐g‐PANI),^[^ ^49^ ^]^ and PSS‐Na^[^ ^51^ ^]^ has been shown to tune the work function of PEDOT:PSS and prevent emission quenching. Alternatively, introducing ultrathin insulating interlayers, e.g., poly vinylpyrollidone (PVP),^[^ ^52^ ^]^ black phosphorous,^[^ ^53^ ^]^ and polyvinylidene fluoride (PVDF),^[^ ^54^ ^]^ has also been shown to improve the device performance of PeLEDs with improved band alignment. Although device performance improvement has been achieved by modifying PEDOT:PSS, it is still not sufficient to ensure long‐term stability and the feasibility of PEDOT:PSS as HTLs for PeLEDs remain a question. In addition to PEDOT:PSS, other conjugated polyelectrolytes (CPEs) have been investigated as HTL for PeLEDs. CPEs are especially attractive because of their tunable energy levels and charge carrier mobility by modifying the polymer backbone and counter ions.^[^ ^55^ ^]^

Covalent small‐molecular and polymeric semiconductors have been used as CTLs for OLEDs, QDLEDs and now PeLEDs (Figure 4c). To date, the use of small‐molecular CTLs has mainly been limited to vapor‐deposited ETLs in the p−i−n devices and HTLs in n−i−p devices,^[^ ^56^, ^57^ ^]^ due to the lack of orthogonal solvents for layer‐by‐layer processing of the CTLs and MHPs. In addition to TPBi,^[^ ^16^, ^18^, ^58^ ^]^ several organic semiconductors, including 4,6‐bis(3,5‐di(pyridin‐3‐yl)phenyl)‐2‐methylpyrimidine (B3PyMPM),^[^ ^13^ ^]^ 1,3,5‐Tri(m‐pyridin‐3‐ylphenyl)benzene (TmPyPb),^[^ ^59^ ^]^ and 2,4,6‐Tris[3‐(diphenylphosphinyl)phenyl]‐1,3,5‐triazine (PO‐T2T),^[^ ^60^ ^]^ have been found to be suitable for high‐performance PeLEDs. The higher electron mobilities and triplet energy, as well as deeper highest occupied molecular orbital levels, enable efficient electron transport, hole blocking, and exciton confinement. Recently, Liao and coworkers showed that the use of PO‐T2T could allow sub‐bandgap turn‐on voltages facilitated by Auger‐assisted charge injection.^[^ ^60^ ^]^ While highly efficient PeLEDs could be obtained with vapor‐deposited ETLs, the rationale behind the choice of ETLs is not well understood and further studies on the interactions between ETLs and MHP layers are needed. Unlike small molecules that could only be processed using vapor deposition, many polymeric CTLs could be solution processed together with MHP layers using orthogonal solvents, particular polymeric HTLs, including poly(9‐vinylcarbazole) (PVK),^[^ ^61^ ^]^ poly(9,9‐dioctylfluorene‐alt‐*N*‐(4‐sec‐butylphenyl)‐diphenylamine) (TFB),^[^ ^21^ ^]^ and poly(*N,N'*‐bis‐4‐butylphenyl‐*N,N'*‐bisphenyl)benzidine (poly TPD).^[^ ^62^ ^]^ The use of polymeric HTLs has afforded NIR PeLEDs with state‐of‐the‐art efficiencies of more than 20%.^[^ ^14^, ^15^, ^63^ ^]^ Due to the low surface energy of pristine polymeric CTLs, treatments are needed to modify the surface properties to reduce the contact angle and enable the formation of uniform MHP thin films with low defect density. These involve O_2_ plasma,^[^ ^62^ ^]^ or insertion of interlayers, such as polyelectrolytes^[^ ^64^, ^65^ ^]^ and LiF.^[^ ^58^ ^]^ Developing cross‐linkable CTLs is another effective strategy well explored in OLEDs to avoid the interfacial mixing between different layers during solution processing of multilayer structures,^[^ ^66^, ^67^ ^]^ which however has not been extensively investigated for PeLEDs to date.^[^ ^68^ ^]^ To deliver ideal organic/polymeric CTLs for PeLEDs, further research is needed to improve the charge carrier mobility and stability, as well as the compatibility with MHP EMLs.

Inorganic CTLs have also been used in PeLEDs, among which metal oxides are of particular interest because of their high mobility, intrinsic chemical stability, and ability to form good band alignment with MHPs. Nickel oxide (NiO_*x*_) as HTL and zinc oxide (ZnO) as ETL are perhaps the two most common cases. Lee et al. demonstrated highly efficient quasi‐2D PeLEDs containing a NiO_*x*_ HTL with EQEs of >14%, in which the use of NiO_*x*_ HTL afforded MHP crystallites with low trap density at the HTL/EML interface.^[^ ^48^ ^]^ The high efficiency was also ascribed to the appropriate band alignment and small hole injection barrier. Moreover, the NiO_*x*_‐based devices showed significantly improved operational stability as compared with PEDOT:PSS‐based devices. Similarly, ZnO nanoparticles have been used as ETLs in PeLEDs. To prevent nonradiative recombination and improve band alignment, interlayers have also been introduced between ZnO/MHP layers, such as polyethylenimine ethyoxylated (PEIE)^[^ ^69^, ^70^ ^]^ and PVP^[^ ^71^ ^]^. The use of ZnO/PEIE ETL has afforded NIR PeLEDs with state‐of‐the‐art performance.^[^ ^14^, ^15^, ^63^, ^72^ ^]^ Hosono and coworkers reported some of the highest luminance from PeLEDs with ZnO‐based ETLs, i.e., 500 000 and 20 000 cd m^−2^ for green and red emissions, respectively. This was realized by tuning the energy band edge positions and electron mobility of ZnO through alloying with Si to achieve excellent charge balance in the EML (Figure 4d).^[^ ^57^ ^]^ Other metal oxides, such as TiO_2_ and SnO_2_, have had limited successes in PeLEDs, due to their deep conduction band minimum with large electron injection barriers.^[^ ^11^, ^73^, ^74^ ^]^ While metal oxides hold great promise for efficient and stable PeLEDs, the high‐temperature annealing needed for their thin‐film formation on MHP layers presents a risk that has to be addressed in future studies. Some of the more notable CTLs are shown in **Table** **1**.

**Table 1:** Examples of CTLs Used in PeLEDs

CTL	Type	Device structure	EQE (%)	Ref
Small molecule				
B3PyMPM	ETL	ITO/PEDOT:PSS/CsPbBr_3_:MABr/PMMA/B3PyMPM/LiF/Al	20.3	[13]
TmPyPb	ETL	ITO/PEDOT:PSS/perovskites/TmPyPb/LiF/Al	1.35	[59]
PO‐T2T	ETL	ITO/PEDOT:PSS/FAPbBr_3_ NCs/PO‐T2T/Ca/Al	13.4	[166]
Polymeric				
PVK	HTL	ITO/PVK/PFI/LCE film (P2m2)/3TPYMB/Liq/Al	2.6	[167]
TFB	HTL	ITO/TFB/LiF/perovskite (5% TPPCl)/TPBi/LiF/Al	19.1	[168]
Poly TPD	HTL	ITO/ZnO/ PEIE/FAPbI_3_/poly‐TPD/MoO_3_/Al	20.2	[169]
Inorganic metal oxide				
ZnO	ETL	ITO/ZnO/PEIE/FACsPbI_3_:TTDDA/TFB/MoO_3_/Ag	19.6	[72]
NiO_x_	HTL	ITO/NiO_*x*_/BA_2_FA_2_PbBr_10_ film/TPBi/LiF/Al	14.6	[48]

In addition to the standard device structure, some unique ones have also been reported for PeLEDs to suppress leakage from pinholes in the emitting MHP layers. Ling et al. used a bipolar polymeric host to reduce charge leakage in MHP nanoplatelets‐based PeLEDs. The application of this host improved the device luminance and EQE by an order of magnitude.^[^ ^75^ ^]^ Shi et al. also showed the use of a new device structure, where the MHP EML was sandwiched between two insulator layers (in this case LiF), which they dubbed insulator−perovskite−insulator (IPI).^[^ ^76^ ^]^ The major advantage of this device structure was shown to be the suppression of charge leakage by the insulating bilayer (Figure 4e). PeLEDs using this architecture displayed several‐fold increase in both luminance and EQE for various 3D MHPs, including MAPbBr_3_, FAPbBr_3_, and CsPbBr_3_. The same device architecture was recently used to fabricate mixed halide blue PeLEDs with a long operational lifetime (*T*_50_) of 300 min.^[^ ^77^ ^]^ Although these novel device architectures are encouraging early steps for MHP‐tailored CTLs and device structures, their application has been limited to EMLs with poor morphology, and investigation into their suitability for a broad type of EML morphology is needed.

The high bipolar charge mobility, tunable energy bandgaps and band edges, and easy processing make MHPs very attractive candidates for CTLs. Our group first demonstrated the use of MAPbCl_3_ as a viable alternative to PEDOT:PSS in solution‐processed PHOLEDs (Figure 4f).^[^ ^78^ ^]^ Further efforts by Adachi's group in using MHPs for OLEDs showed the use of up to micron‐thick MHP CTLs with state‐of the art performance.^[^ ^79^ ^]^ The ability to obtain thickness‐independent mobility from MHP CTLs makes them invaluable in large‐area fabrication of OLEDs with reduced performance variation due to thickness nonuniformity. More recently, Park and colleagues have shown the use MAPbCl_3_ as HTL to obtain green emitting PeLEDs with a peak EQE of over 5%.^[^ ^80^ ^]^ To further explore the potential of MHPs as CTLs in PeLEDs, effective approaches have to be develop to enable the preparation of multilayer MHP heterojunction structures and prevent ion migration between different MHP layers. Atomic layer deposition for the formation of an ultrathin insoluble layer on top of an MHP HTL followed by solution processing of a MHP EML could be one of the solutions.^[^ ^81^, ^82^ ^]^

Although much progress has been recorded on improving the compatibility of existing CTLs with PeLEDs, several issues and challenges remain to be addressed. Future efforts should be directed to designing highly stable CTLs with energy band edges specifically tailored to distinct MHPs. Tuning the charge mobilities could also lead to improved charge balance and device efficiencies. Moreover, fundamental understanding of the interactions in the CTL/MHP interfaces, as well as the behaviors of charges and excitons in these interfaces, is needed. Attention should also be paid to the processability of CTLs to ensure facile fabrication of multilayer heterojunction structures with MHP EMLs.

#### 2.1.3 Efficiency Roll‐Off

**Figure 5:**
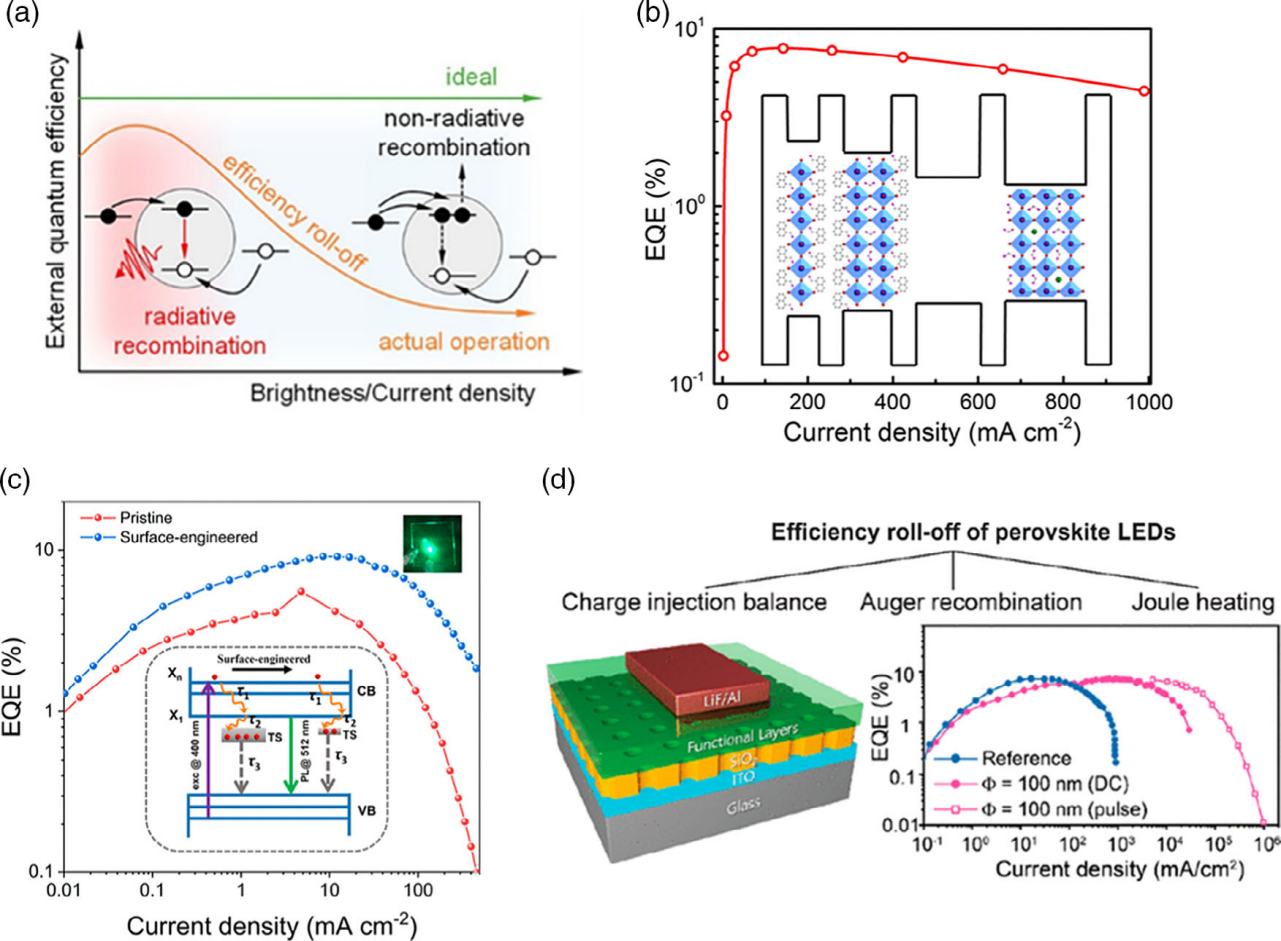
a) Diagram which exemplifies efficiency roll‐off under ideal and actual LED device operation. The EQE decreases with increasing brightness and current density under actual operating conditions, b) reduced efficiency roll‐off in MQW PeLEDs by controlling quantum well width, c) EQEs of PeLEDs based on pristine and surface‐engineered MHP NCs; inset shows the photophysical processes in pristine and surface‐engineered MHP NCs, d) left: device structure of nanopatterned PeLED to reduce Joule heating; right: EQEs of reference, nanopatterned device under DC and pulse operation. a) Reproduced with permission.^[^ ^153^ ^]^ Copyright 2020, American Chemical Society. b) Reproduced with permission.^[^ ^86^ ^]^ Copyright 2018, American Chemical Society. c) Reproduced with permission.^[^ ^87^ ^]^ Copyright 2020, American Chemical Society. d) Reproduced with permission.^[^ ^85^ ^]^ Copyright 2020, American Chemical Society.

Efficiency roll‐off is the decline of device efficiency with increasing brightness and current density (**Figure** **5**a). For displays and lighting applications requiring operation at high brightness, severe efficiency roll‐off can be problematic. While efficiency roll‐off is a common challenge in all types of LEDs, their causes could be distinct for different kinds of LEDs. Considerable effort has been applied on gaining fundamental understanding of the underlying causes of efficiency roll‐off, as well as methods to reduce efficiency roll‐off to improve the device performance of OLEDs, QDLEDs, and now PeLEDs. In general, efficiency roll‐off is considered to be caused by a combination of several different phenomena, including Joule heating at high current density,^[^ ^46^ ^]^ electric field‐induced emission quenching,^[^ ^83^ ^]^ and charge imbalance.^[^ ^84^ ^]^ Experimental methods to study efficiency roll‐off behaviors involve measuring the electroluminescence and photoluminescence intensities of an LED, sometimes alongside measurements of the transient photoluminescence decay and quantum‐confined Stark effect.^[^ ^83^ ^]^

Recent studies have found that efficiency roll‐off in PeLEDs is caused by a combination of Auger loss, Joule heating, and charge imbalance.^[^ ^85^ ^]^ Great successes in preventing nonradiative Auger recombination in PeLEDs have been achieved in recent years. For instance, Huang and coworkers have demonstrated the formation of quantum wells with increased well thickness to suppress the efficiency roll‐off caused by Auger recombination in multiple‐quantum‐well (MQW) PeLEDs (Figure 5b).^[^ ^84^, ^86^ ^]^ Yao et al. demonstrated surface engineering of MHP NCs via a hybrid ligand passivation approach for the suppression of nonradiative Auger recombination in MHP thin films (Figure 5c).^[^ ^87^ ^]^ To minimize the effects of Joule heating, Rand's team combined several thermal management strategies, including doping charge transport layers, optimizing device geometry, and attaching heat spreaders and sinks, for high‐performance PeLEDs with greatly reduced efficiency roll‐off.^[^ ^88^ ^]^ Balancing charge injection is crucial to achieve not only high EQEs, but also low efficiency roll‐off in PeLEDs. Heremans and coworkers demonstrated PeLEDs with enhanced EQE, low efficiency roll‐off, and long operational lifetime, by tuning the balance of electron/hole transport in a mixed 2D/3D MHP EML.^[^ ^89^ ^]^ The balancing of charge carrier transport/injection leads to optimized spatial charge carrier distribution, avoiding excess carrier leakage at high current densities. In this system, exciton confinement in the mixed 2D/3D EML also helps reduce nonradiative recombination. Balancing charge injection could also be achieved using appropriate electrode interface layers.^[^ ^90^ ^]^ Recently, a multidimensional design of suppressing efficiency roll‐off by a combination of balancing charge injection, reducing nonradiative Auger recombination, and mitigating Joule heating has been reported by Lin's group (Figure 5d).^[^ ^85^ ^]^ First, a step‐wise “energy ladder” was designed to balance the electron and hole transport and reduce emission quenching. Second, MHP EMLs were optimized to possess reduced Auger recombination rates and improved carrier mobility. Third, glass substrates were replaced by sapphire substrates to better dissipate Joule heat. Such cocktail approaches to address the issues of nonradiative recombination, Joule heating, and charge imbalance simultaneously are highly promising and worthy of further investigation. Meanwhile, a number of strategies that have been used in OLEDs and QDLEDs to reduce the efficiency roll‐off have the potential to be adapted for PeLEDs. For instance, nanostructures with surface plasmon resonance effect might be integrated with PeLEDs for suppression of efficiency roll‐off.^[^ ^91^, ^92^, ^93^ ^]^

#### 2.1.4 Light Outcoupling Efficiency

The EQE of an LED is a product of the IQE and light outcoupling efficiency (*η*_out_). With IQEs of near 100% being achieved in PeLEDs, realizing high EQEs would rely on the improvement of light outcoupling or the extraction of generated photons. Light outcoupling management has been extensively investigated in OLEDs, QDLEDs, and conventional inorganic LEDs for decades. It is well accepted that the extractions of the generated photons in these devices with planar geometry are constrained due to the high refractive indices of the active layers and substrates. Various classical lossy modes responsible for the poor light outcoupling include surface plasmon polaritons, waveguide modes, substrate modes, and electrode absorption.

**Figure 6:**
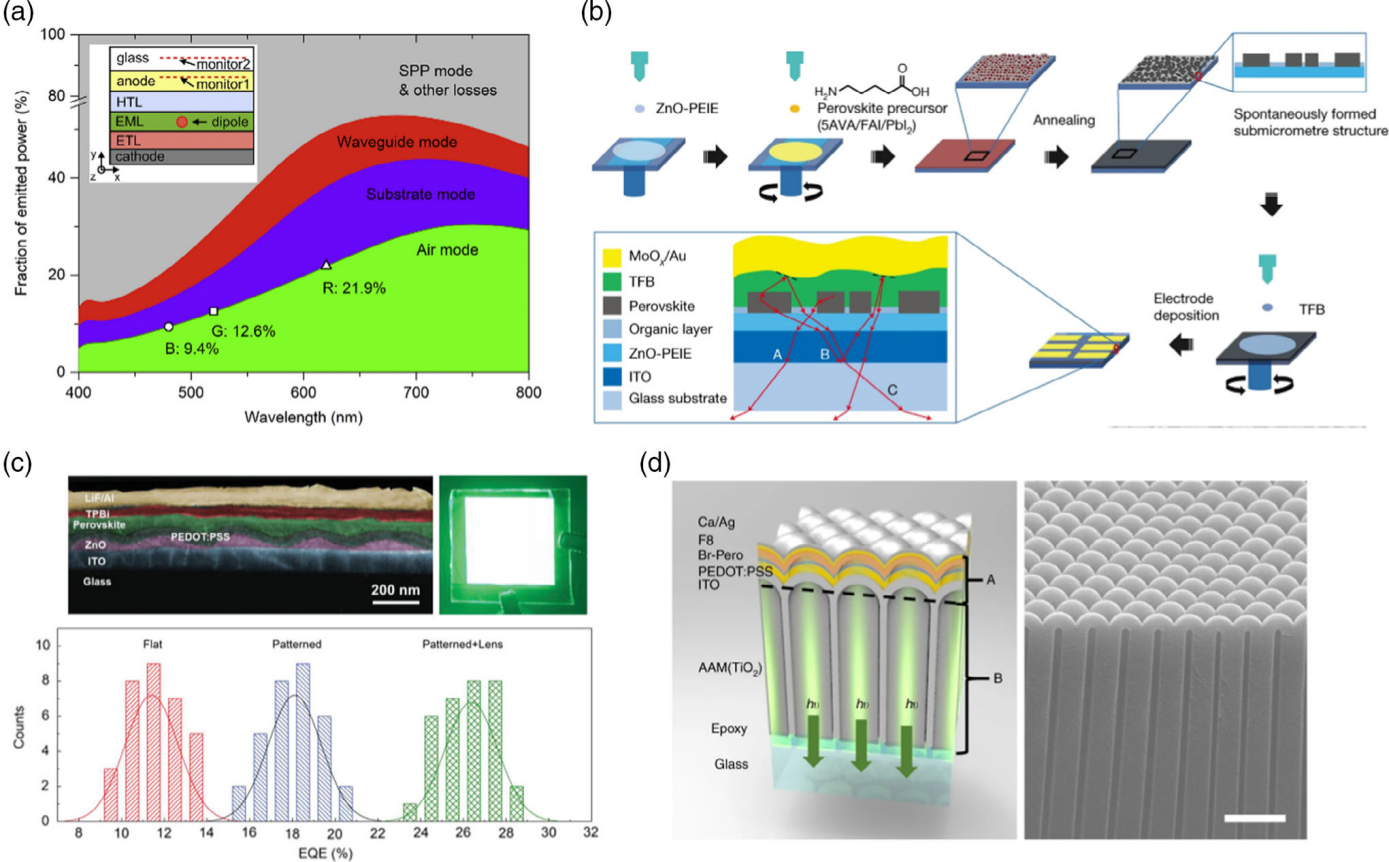
a) Fractions of various mode powers of planar PeLEDs with an isotropic dipole orientation as a function of dipole emitting wavelength; inset: schematic illustration of device structure, b) solution‐processed MHP thin films with spontaneous formation of submicrometre‐scale structures, c) top: cross‐sectional scanning electron microscopy (SEM) image of CsPbBr_3_ PeLED with imprinted nanostructures and PeLED under operation; bottom: EQEs of flat, patterned, and patterned with lens PeLEDs, and d) left: device structure of PeLED on nanophotonic substrate; right: SEM image of nanophotonic substrate. a) Reproduced with permission.^[^ ^95^ ^]^ Copyright 2018, Elsevier. b) Reproduced with permission.^[^ ^14^ ^]^ Copyright 2018, Springer Nature. c) Reproduced with permission.^[^ ^98^ ^]^ Copyright 2019, Wiley‐VCH. d) Reproduced under the terms of CC‐BY 4.0 license.^[^ ^100^ ^]^ Copyright 2019, The Authors, published by Springer Nature.

While possessing great similarity with OLEDs and QDLEDs, PeLEDs have some unique properties that affect the light outcoupling efficiency, as a result of high refractive indices and photon reabsorption within the EMLs.^[^ ^94^ ^]^ Recent theoretical and experimental studies on PeLEDs have generated a clear picture on lossy modes in PeLEDs. For instance, Tang and coworkers provided a quantitative analysis of power loss channels and light outcoupling manipulation in PeLEDs through systematic theoretical simulations (**Figure** **6**a).^[^ ^95^ ^]^ Greenham and coworkers analyzed the role of photon recycling in assisting light extraction from PeLEDs and proposed schemes incorporating reduced electrode area and various filtering structures to maximize the benefit of photon recycling in PeLEDs.^[^ ^96^ ^]^

Taking the similarity and difference between OLEDs and other types of LEDs into account, many successful light outcoupling management approaches in OLEDs and QDLEDs have been adapted for PeLEDs with appropriate modifications. Those approaches include 1) controlling the microstructure and thickness of the active layer, 2) introducing outcoupling structures between the active layer and transparent electrodes, and 3) adding outcoupling structures on top of the transparent substrate. Here, a few representative works are highlighted. Huang and coworkers applied a simple molecular engineering approach, introducing amino acid additives into the MHP precursor solutions, to create microstructures inside the MHP layer to improve the light extraction (Figure 6b).^[^ ^14^ ^]^ Rand and coworkers adopted thin MHP layers in the range of 35–40 nm to reduce waveguiding for enhanced light outcoupling.^[^ ^97^ ^]^ Tang and coworkers embedded bioinspired moth‐eye nanostructures at the front electrode/MHP interface to dramatically improve light extraction, resulting in green emitting PeLEDs with an EQE exceeding 28% (Figure 6c).^[^ ^98^ ^]^ Wang and coworkers applied a top‐emission device structure with microcavity effect to improve the light extraction and achieve high‐efficiency PeLEDs with an EQE of 20.2%. The microcavity was formed using a total‐reflection gold bottom electrode and a semitransparent gold top electrode in a simple top‐emission (TE) LED device structure.^[^ ^99^ ^]^ Similarly, Fan and coworkers demonstrated a two‐step light extraction process involving nanodome light couplers and nanowire optical antennas on the nanophotonic substrate for efficient PeLEDs with an EQE of 17.5%, which is around twice of the record for the planar device based on this material system.^[^ ^100^ ^]^ Fan and coworkers have a comprehensive review on the light outcoupling management in PeLEDs with lessons learned from the past.^[^ ^94^ ^]^

While remarkable achievements have been obtained in light outcoupling management of PeLEDs during the last couple of years, there is still a large gap between the state‐of‐the‐art outcoupling efficiency and theoretical maximum. As light outcoupling is controlled by multiple factors, integrated strategies for synergistic light outcoupling management remain to be developed. Through simultaneous optimization of the refractive index and film thickness of layer stacks, control of the transition dipole orientation of MHP emitters, implementation of photonic structures into the active layers, and utilization of proper transparent electrodes and substates, we expect that light outcoupling efficiency of PeLEDs will soon catch up with those of OLEDs and QDLEDs.

### 2.2 Device Operational Stability

The operational stability of PeLEDs is one of the key issues remaining to be addressed before their commercial use. Operational lifetime of an LED could be determined by measuring the elapsed time for the electroluminescence to decay by 50% (*T*_50_), 80% (*T*_80_), or 90% (*T*_90_) of its initial intensity. For commercially available OLEDs, *T*_50_ (at initial luminance of 1000 cd m^−2^) of around 1 million h has been achieved for red and green PhOLEDs. However the longest lifetime reported to date for PeLEDs is only few hundreds of hours,^[^ ^101^, ^102^ ^]^ far from the required value for practical applications of PeLEDs. With device efficiency of PeLEDs approaching those of OLEDs and QDLEDs, stability of PeLEDs has received more research attention during the last few years.^[^ ^103^, ^104^ ^]^ Experimental and theoretical efforts have shed some light on the degradation mechanisms of PeLEDs, although a comprehensive fundamental understanding is still lacking. It remains a question if it is theoretically possible to deliver PeLEDs with operational lifetime as long as that of OLEDs and QDLEDs. According to current understanding, degradation of PeLEDs is mainly caused by ion migration, electrochemical, and interfacial reactions, given that the devices are well encapsulated with negligible effect from the ambient environment. All these issues are associated with the ionic nature of MHPs, which OLEDs and QDLEDs do not share.^[^ ^105^, ^106^ ^]^ Therefore applying the same approaches used to address the stability issues of OLEDs and QDLEDs could not completely solve the problems of PeLEDs, although the same research methodologies could be applied to study the degradation mechanisms, improve material properties, and optimize device structures.

So and coworkers recently presented a comprehensive review on the degradation mechanism of PeLEDs and potential solutions,^[^ ^104^ ^]^ in which they have outlined three processes that could be responsible for the degradation of PeLEDs, including ion migration, electrochemical reaction, and interfacial processes. Photo‐ and electric field‐induced ion migration has been the focus of study in MHP solar cells (PSCs) for quite some time. Similarly, this ion migration also leads to PeLED degradation through migration of ions in the perovskite emitter to electrode interfaces with the formation of defect species and complexes that induce the formation of midgap states, such as Pb^0^ interstitials, as well as migration into CTLs and electrodes that modify the charge transport and injection properties of these components. Interestingly, ion migration and annihilation of Frenkel defects temporarily increase electroluminescence and device efficiency; however, this luminance overshoot inevitably leads to fast degradation of PeLEDs. Lee and coworkers recently showed that luminance overshoot can be mitigated through effective suppression of ion migration.^[^ ^107^ ^]^ Moreover, electrochemical reactions at electrode interfaces that lead to the formation of insulating metal halide salts create charge injection barriers and shift recombination zones resulting in emission quenching and device degradation. These processes are thought to be irreversible as the decomposition of MHP grains can be accompanied by permanent halide or A^+^ cation loss in the form of halogen gases or methylamine,^[^ ^108^ ^]^ respectively. Finally, reactions at CTL/EML interfaces, such as In^3+^ migration from ITO electrodes that result in PL quenching, can also lead to poor device stability. Joule heating of PeLEDs has also been identified as a cause for device degradation, where improved thermal management techniques lead to enhanced device stability.^[^ ^88^ ^]^

**Figure 7:**
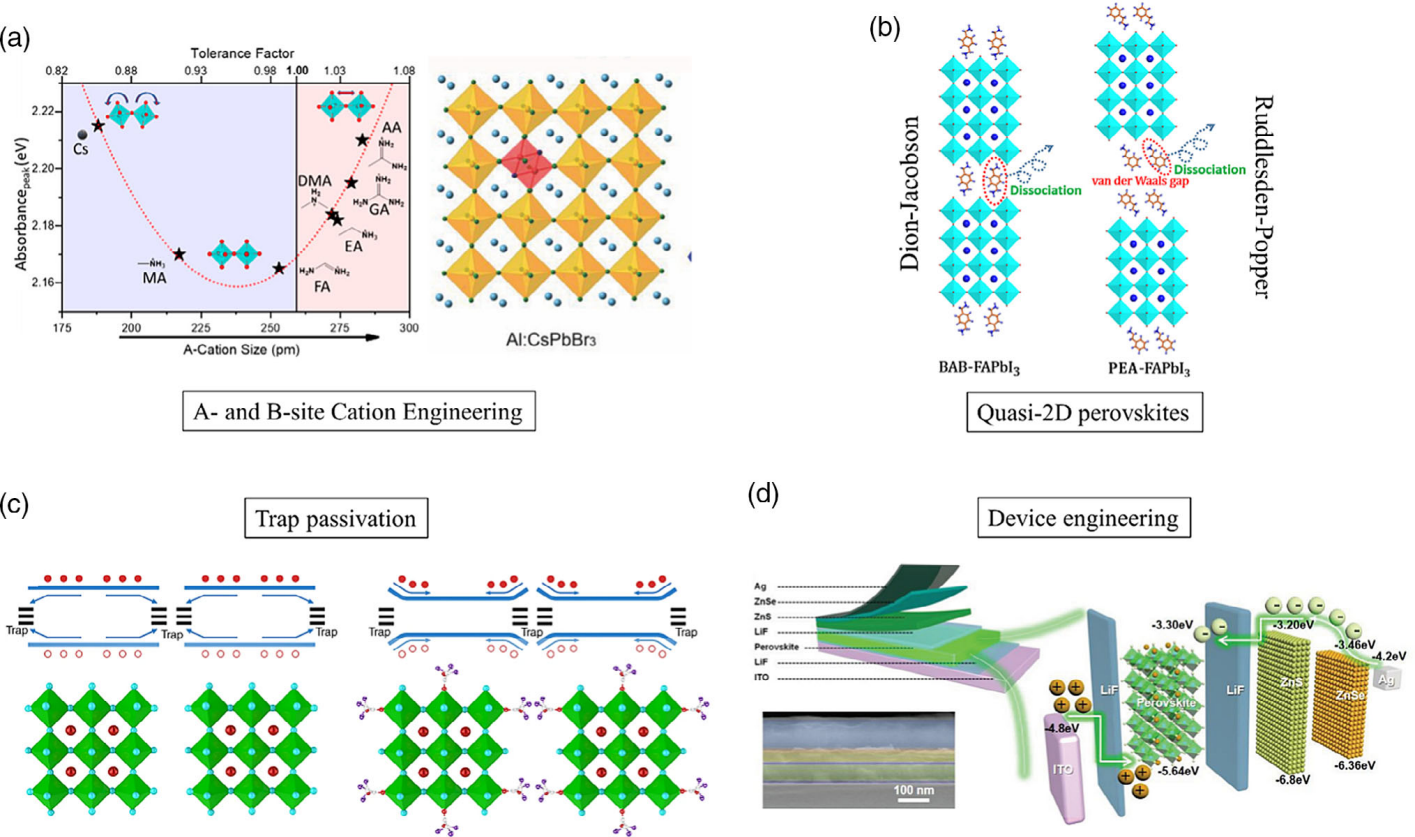
Strategies to improve PeLED operational stability: a) A‐ and B‐site substitution, b) formation of quasi‐2D perovskites with Dion−Jacobson and RP phases, c) surface defect and grain boundary passivation, d) device structure and charge transport engineering for suppression of ion migration. a) A‐site: Reproduced with permission.^[^ ^162^ ^]^ Copyright 2020, American Chemical Society. B‐site substitution: Reproduced under the terms of the CC‐BY 4.0 license.^[^ ^163^ ^]^ Copyright 2017, The Authors, published by Wiley‐VCH. b) Reproduced with permission.^[^ ^109^ ^]^ Copyright 2019, American Association for the Advancement of Science. c) Reproduced under the terms of the CC‐BY 4.0 license.^[^ ^101^ ^]^ Copyright 2019, The Authors, published by Springer Nature. d) Reproduced with permission.^[^ ^102^ ^]^ Copyright 2020, Wiley‐VCH.

To improve the stability of PeLEDs, many strategies have been developed to stabilize the MHP phase with suppressed ion migration, including 1) compositional engineering of A‐ and B‐ sites in 3D ABX_3_ MHPs (**Figure** **7**a), 2) utilizing quasi‐2D MHPs (Figure 7b), 3) surface and grain defect passivation (Figure 7c), and 4) applying appropriate CTLs and electrodes (Figure 7d). As the degradation processes of PeLEDs are multidimensional, combining these strategies rather than relying on individual ones is needed to tackle the stability issues. Several notable works are highlighted here. Our group developed quasi‐2D MHP/PEO composite thin films for red PeLEDs with great spectral and operational stability. The enhanced operational stability in air, with the electroluminescence intensity dropping at about 20% after 4 h of continuous operation, is attributed to the high stability of cesium‐based quasi‐2D MHPs and uniform pinhole‐free passivated polymer/MHP composite thin films.^[^ ^40^ ^]^ Similarly, Gao and coworkers developed MHP−molecule composite thin films, consisting of in situ‐formed high‐quality MHP NCs embedded in an electron‐transport molecular matrix, with which efficient and stable PeLEDs with a peak EQE of 17.3% and half‐lifetime of 100 h at a constant current density of 20 mA cm^−2^ (initial radiance of 15 W m^−2^ sr^−1^) were demonstrated.^[^ ^42^ ^]^ Ning and coworkers demonstrated the use of Dion−Jacobson (DJ) quasi‐2D MHPs for stable PeLEDs with a half‐lifetime over 100 h, which is almost two orders of magnitude longer than that of PeLEDs based on Ruddlesden−Popper (RP) quasi‐2D MHPs. Theoretical simulation studies suggested that the DJ quasi‐2D MHP is more robust with a higher molecular dissociation energy than the RP structural one.^[^ ^109^ ^]^ Lee and coworkers demonstrated PeLEDs based on 3D/2D hybrid MHPs with 21 times longer operational lifetime than PeLEDs based on 3D MHPs. The 3D/2D hybrid MHPs were prepared by adding benzylamine to MAPbBr_3_, which enabled crystallization of 2D MHPs without destroying the 3D phases. The presence of benzylammonium in the MHP lattice suppresses the formation of deep trap states and ion migration.^[^ ^107^ ^]^ A similar 3D/2D hybrid approach was reported by Yang and coworkers, in which surface2D/bulk 3D heterophased core–shell‐like MHP nanograins were strategically designed to afford stable PeLEDs with an operational half‐lifetime of more than 200 h.^[^ ^110^ ^]^ Rogach and coworkers used cesium trifluoroacetate as a precursor for CsPbBr_3_, which improved the photophysical properties. They surmised trifluoroacetate anions can effectively passivate grain boundaries and reduce the trap density. PeLEDs based on this strategy displayed a *T*_50_ of 250 h at an initial luminance of 100 cd m^−2^.^[^ ^101^ ^]^ Wu and coworkers developed an all‐inorganic strategy involving an IPI device structure and cascade ZnS−ZnSe electron transport layers for stable PeLEDs. With enhanced charge injection efficiency and suppressed electric field‐induced ion migration, PeLEDs based on (Cs_1−*x*_Rb_*x*_)_1−*y*_K_*y*_PbBr_3_ exhibited an EQE of 11.05% and high operational stability with a *T*_50_ of about 255 h at an initial luminance of 120 cd m^−2^.^[^ ^102^ ^]^

The improvement in the stability of PeLEDs, with the operational lifetime increasing from only a few seconds to, nowadays, a few hundred hours, is highly encouraging. Meanwhile, achievements in realizing highly stable PSCs^[^ ^111^ ^]^ and luminescent MHP NCs^[^ ^44^ ^]^ suggest that MHPs could have high photostability and thermal stability. According to the guidelines obtained from recent studies, confining defect‐free MHP phases in nanodomains, either quasi‐2D phases or core−shell NCs with surface passivation, to completely suppress the ion migration is perhaps the most promising approach to addressing the stability issues. With deactivation of ion migration, MHP emitters could essentially be treated as semiconducting NCs and all the successful strategies used for stable QDLEDs could be adopted for PeLEDs.

### 2.3 Toxicity

The toxicity of Pb‐based MHPs is one of the major challenges that has to be addressed before MHP‐based devices can be commercialized in consumer products. Pb is a regulated toxic substance in most of the world with the maximum contaminant level (MCL) of Pb in water set as 0.015 mg L^−1^ in the US.^[^ ^112^ ^]^ The easier leaching of Pb^2+^ by water in lead halide perovskites as compared with that of metallic Pb or other Pb compounds makes the toxicity of lead halide perovskites more concerning. A recent study on the extraction of Pb^2+^ from PSCs conducted via dynamic leaching tests (DLT) has shown that a non‐negligible amount of Pb^2+^ can be extracted from the devices.^[^ ^113^ ^]^ To address the toxicity of lead halide perovskites, tremendous research efforts have been done on the development of lead‐free MHPs, such as tin halide perovskites, for various types of optoelectronic devices, including PeLEDs.^[^ ^114^, ^115^, ^116^, ^117^ ^]^ However, no lead‐free PeLEDs with comparable device performance as lead‐based PeLEDs have been demonstrated to date. Developing effective strategies to suppress the extraction of Pb^2+^ from lead halide perovskites represents another approach to addressing the Pb toxicity issue if lead halide perovskites cannot be replaced.

#### 2.3.1 Lead‐Free MHPs

**Figure 8:**
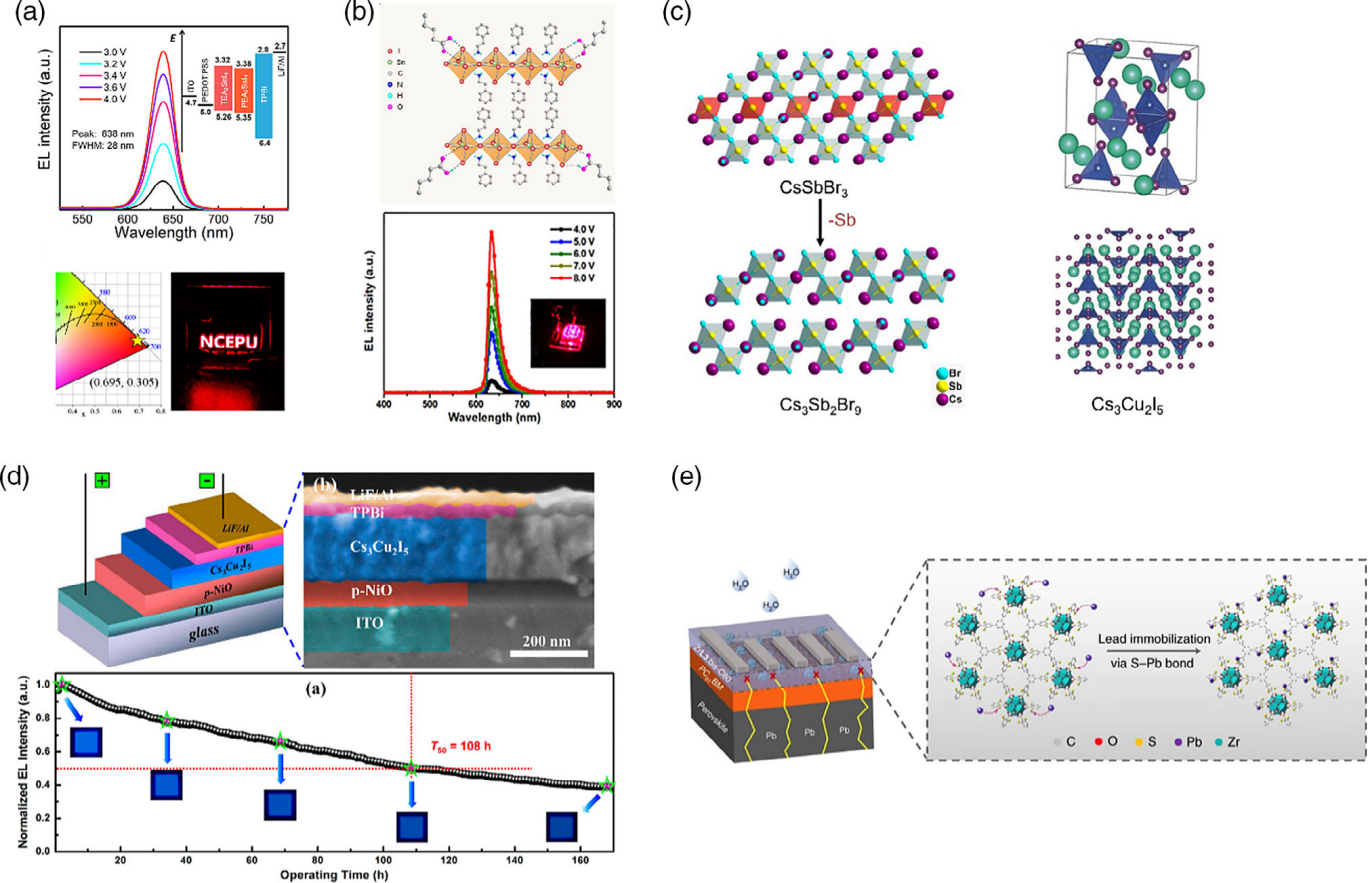
a) Top: electroluminescence spectra of the TEA_2_SnI_4_‐based device. The inset shows the energy band diagram of the 2D tin‐based PeLED devices; bottom: Commission Internationale de l'éclairage coordinates of PeLEDs based on TEA_2_SnI_4_ and a photograph of a PeLED with the North China Electric Power University logo, b) top: illustration of crystal structure of PEA_2_SnI_4_ containing valeric acid; bottom: electroluminescence spectra and picture of PeLED under operation, c) left: removal of every third Sb layer along the ⟨111⟩ direction of the perovskite structure results in the 2D layered Cs_3_Sb_2_Br_9_ structure, right: the crystal structure of Cs_3_Cu_2_I_5_, d) top: schematic structure and cross‐sectional SEM image of the Cs_3_Cu_2_I_5_ NCs‐based LEDs; bottom: evolution of the emission intensity of the device over the running time, e) Pb^2+^ capture by the 2D MOF ZrL3 in PSCs. a) Reproduced with permission.^[^ ^122^ ^]^ Copyright 2020, American Chemical Society. b) Reproduced with permission.^[^ ^124^ ^]^ Copyright 2020, American Association for the Advancement of Science. c) Left: Reproduced with permission.^[^ ^165^ ^]^ Copyright 2017, American Chemical Society. Right: Reproduced with permission.^[^ ^164^ ^]^ Copyright 2018, Wiley‐VCH. d) Reproduced with permission.^[^ ^130^ ^]^ Copyright 2020, American Chemical Society. e) Reproduced with permission.^[^ ^132^ ^]^ Copyright 2020, Springer Nature.

Tin halide perovskites are one of the most investigated lead‐free MHPs to date. Although Sn^2+^ shares a similar electronic structure and ionic radius as Pb^2+^, which enables it to successfully form 3D, quasi‐2D, and 2D MHPs with narrow emissions, the application of tin halide perovskites for PeLEDs has been fraught with challenges. Unlike lead halide perovskites with near‐unity PLQEs easily achieved, tin halide perovskites, developed to date, possess low PLQEs with the highest recorded around 21%.^[^ ^118^ ^]^ Other issues for tin halide perovskites include easy oxidation of Sn^2+^ to Sn^4+^ that leads to p‐type doping and metallization, low formation energy of Sn^2+^ vacancies that create mid‐bandgap trap states, high defect density that introduces nonradiative recombination, and fast crystallization with poor film morphology. Various strategies have been developed to overcome these issues to produce tin‐based PeLEDs with moderate performance. In 2016, CsSnI_3_ NIR PeLEDs with an EQE of 3.8% were demonstrated using antisolvent‐assisted crystallization to improve film morphology and reduce trap density.^[^ ^119^ ^]^ To avoid the oxidation of Sn^2+^, quasi‐2D and 2D tin halide perovskites are preferred over 3D ones. Wang et al. showed tin‐based multiple quantum well thin films with excellent resistance to oxidation and PLQE of 18%, with which NIR PeLEDs with EQEs of 3% were demonstrated.^[^ ^120^ ^]^ Similarly, the use of Sn^2+^ 2D MHPs, such as PEA_2_SnI_4_^[^ ^121^ ^]^ and TEA_2_SnI_4_,^[^ ^122^ ^]^ has been shown to limit Sn^2+^ oxidation in color pure red LEDs with EQEs of 0.3 % and 0.62 %, respectively (**Figure** **8**a). Antioxidative agents, such as Naphtol sulphonic acid, were recently reported to effectively suppress the oxidation of Sn^2+^ and provide trap passivation for red PeLEDs with EQEs of 0.72%.^[^ ^123^ ^]^ Sargent et al. recently showed the use of valeric acid to reduce Sn^2+^ oxidation and defect density, achieving color pure red PeLEDs with an EQE of 5% (Figure 8b).^[^ ^124^ ^]^

Manganese has also been used in MHPs as a dopant system to enhance and stabilize PeLEDs. For instance, Congreve et al. demonstrated the incorporation of Mn^2+^ in mixed halide MHPs leading to improved PLQY and device performance.^[^ ^125^ ^]^ Similarly, Ehrler and colleagues recently showed that the incorporation of a small amount of Mn^2+^ in quasi‐2D PeLEDs improved their operational stability through the increase of the ion‐migration activation energy.^[^ ^126^ ^]^ Alternatively, by doping Mn^2+^ ions in 2D perovskites, Wang et al. have shown yellow emitting LEDs with emissions coming from the Mn^2+^ ions.^[^ ^127^ ^]^ In these examples, the amount of Mn^2+^ ions used is very low and, thus, these strategies are unlikely to lead to the complete replacement of Pb^2+^ and will not address the toxicity issue. Similarly, 0D systems based on Mn^2+^ could also lead to efficient LEDs as previously shown by Xu and coworkers, although the emission characteristics are significantly different from those of PeLEDs.^[^ ^128^ ^]^ In other words, these LEDs with emissions from Mn^2+^ ions are no longer considered as PeLEDs.

Alternatively, heterovalent substitution of Pb^2+^ by other metal ions, such as Cu^+^, Sb^3+^, and Bi^3+^, for the formation of perovskite‐related materials with A_3_B_2_X_9_ and A_3_B_2_X_5_ structures (Figure 8c), has been investigated, which could exhibit great stability and high PLQEs. For instance, Cs_3_Sb_2_X_9_ NCs with tunable emissions from violet to red have been developed to exhibit high PLQEs of up to 51.2%. LEDs based on Cs_3_Sb_2_Br_9_ were fabricated to exhibit a UV emission at 408 nm with an EQE of 0.206 % and an operational lifetime (*T*_90_) of 6 h.^[^ ^129^ ^]^ The relatively high stability of Cs_3_Sb_2_Br_9_ LEDs as compared with CsPbBr_3_ ones is attributed to minimal joule heating and large halide diffusion barrier. Recently, blue LEDs (≈445 nm) based on Cs_3_Cu_2_I_5_ NCs were demonstrated with an EQE of ≈1.12% and a record half‐lifetime of more than 100 h (Figure 8d).^[^ ^130^ ^]^ While these lead‐free perovskite‐related materials provide an alternative avenue to realize stable LEDs, their optical and electronic properties remain to be improved to achieve high‐performance LEDs. Unlike ABX_3_ MHPs with narrow emissions from free excitons, these lead‐free perovskite‐related materials exhibit broadband emissions with large Stokes shifts from self‐trapped excitons. Therefore, developing stable perovskite‐related materials with direct band narrow emissions is of great interest and worthy of further exploration.

#### 2.3.2 Lead Leakage Suppression

Another strategy to limit the effects of Pb toxicity is to suppress its leakage from MHP devices. In this respect, a few recent studies have been conducted on the suppression of Pb^2+^ leakage from PSCs.^[^ ^131^, ^132^, ^133^, ^134^, ^135^ ^]^ For instance, Qi and colleagues showed the use of self‐healing epoxy resin as encapsulation agent to reduce the Pb^2+^ leakage amount by a factor of 375 as compared with glass encapsulation.^[^ ^131^ ^]^ Jen and coworkers used a 2D metal−organic framework (MOF) to improve the stability of PSCs and suppress lead leakage by forming water‐insoluble solids (Figure 8e).^[^ ^132^ ^]^ These studies suggest that proper encapsulation could significantly minimize the Pb^2+^ leakage for MHP devices. In addition to limiting Pb^2+^ leakage, device encapsulation also plays a critical role in device stability. On device Pb^2+^ sequestration has also been demonstrated in PSCs using phosphonic acid‐based chelating agents that are soluble only in certain polar solvents. The strong binding of Pb^2+^ to these chelators reduces its leakage greatly. Moreover, during water‐soaking experiments, the Pb^2+^‐absorbing films did not dissolve but only swelled, making them suitable for a variety of weather conditions.^[^ ^136^ ^]^ Although investigations on the Pb^2+^ leakage in PeLEDs have not been reported to date, the resulting leakage amount is expected to be much less than that in PSCs, provided that less amounts of MHPs are used in PeLEDs with lower thicknesses. Nonetheless, the leakage extent of Pb in PeLEDs should be assessed.

### 2.4 Processing and Patterning of PeLEDs

While efforts on achieving highly efficient and stable PeLEDs remain the major research focus, developing reliable processing and patterning techniques that are compatible with industrial‐scale manufacturing is critical toward commercialization of PeLEDs. Indeed, most of the processing and patterning techniques used for preparation of OLEDs and QDLEDs could be adapted for PeLEDs, e.g., inkjet printing, lithography, vapor deposition, etc.

**Figure 9:**
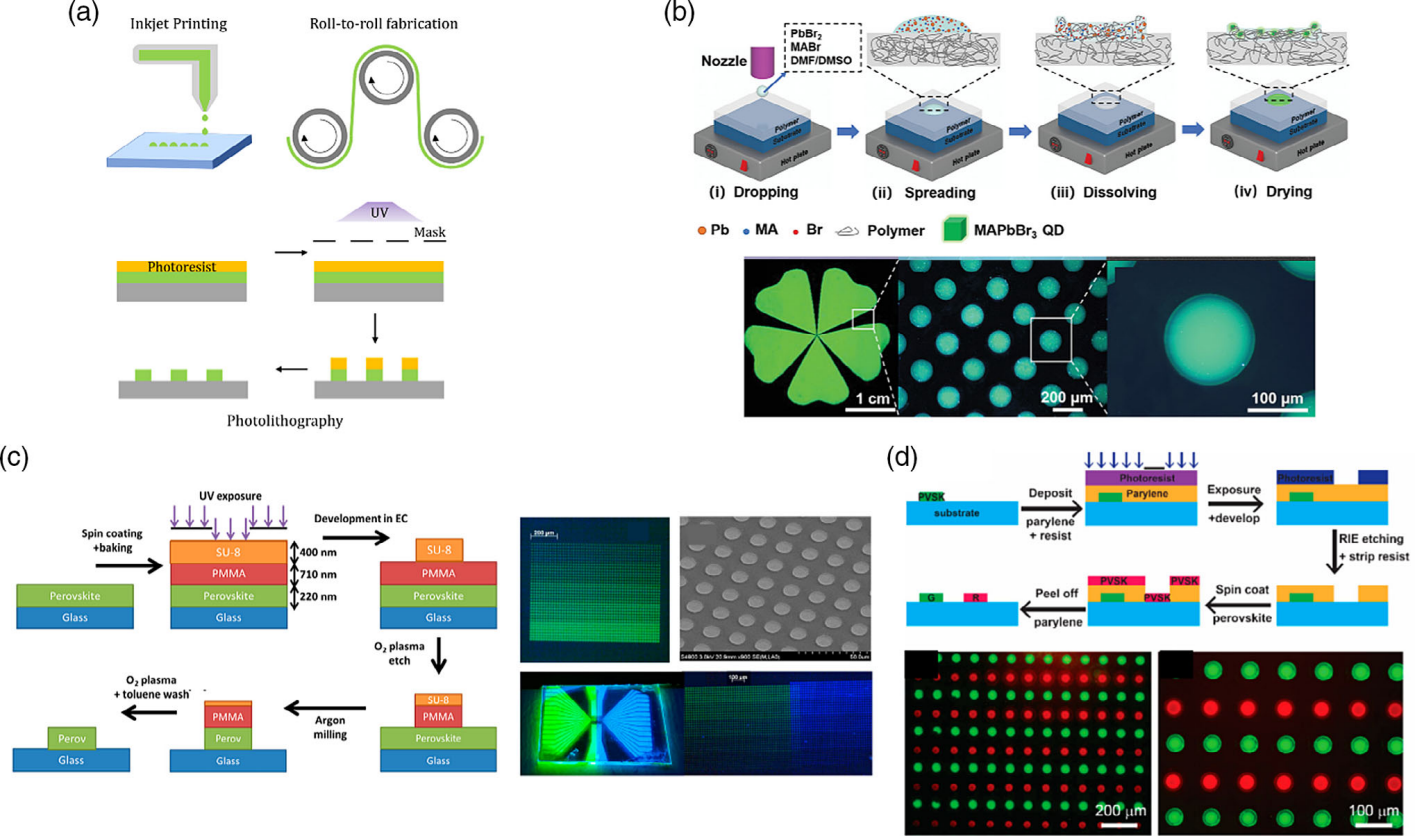
a) Schematic showing inkjet printing, roll‐to‐roll fabrication, and photolithographic processes, b) top: schematic of in situ inkjet printing process; bottom: inkjet‐printed film under UV and a dark‐field microscope, c) left: top−down photolithographic process; right: patterned films under UV and a scanning electron microscope, d) top: schematic fabrication procedures of multicolor MHP patterns; bottom: fluorescent microscope image of green and red MHP circles with a diameter of 50 μm. b) Reproduced with permission.^[^ ^140^ ^]^ Copyright 2019, Wiley‐VCH. c) Reproduced with permission.^[^ ^143^ ^]^ Copyright 2019, American Chemical Society. d) Reproduced with permission.^[^ ^144^ ^]^ Copyright 2020, American Chemical Society.

One of the most attractive features of MHPs is their solution processability. As a highly versatile method, solution processing is cost effective and capable of mass producing large‐area thin films. The ability to synthesize MHPs in situ, through the crystallization of MHP grains on‐substrate, or ex situ, where colloidal MHP NCs are synthesized off‐substrate and later deposited, affords high flexibility of processing PeLEDs. Solution processing techniques can be categorized as those that enable patterning and those that do not. Of those that enable patterning, inkjet printing, lithography, and screen printing are the most widely used in manufacturing, whereas spin coating, doctor blading, slot‐die coating, spray coating, and roll‐to‐roll fabrication are more suitable for large‐area devices that do not require intricate patterning (**Figure** **9**a).^[^ ^137^ ^]^

Recently, Hermerschmidt et al. demonstrated inkjet‐printed PeLEDs with performance comparable with spin‐coated devices, using KCl‐doped PEDOT:PSS HTL to template the growth of MHP crystals. To ensure the formation of smooth and pinhole free thin films, the MAPbBr_3_ precursor solutions were blended with PEG to control the rheological properties.^[^ ^138^ ^]^ Inkjet solutions for PeLEDs could also be prepared using MHP NCs, with which electroluminescent displays with a pixel density of 120 pixels per inch were demonstrated by Peng et al.^[^ ^139^ ^]^ A few other reports have also explored the deposition of MHP NCs and single crystals through inkjet printing using polymer substrates^[^ ^140^ ^]^ polymer matrices to modify the rheological properties of the inks^[^ ^141^ ^]^ and in‐polymer crystal growth (Figure 9b).^[^ ^142^ ^]^ Top−down photolithography has also been used for large‐scale, high‐resolution patterning of MHP thin films, which were previously thought to be infeasible due to incompatibility of MHP films with the solvents used during lithographic processes. Recently, Samuel and coworkers demonstrated photolithography on MHPs using commercial resists with only minor modifications to the standard lithography procedures.^[^ ^143^ ^]^ With the use of a mask aligner or stepper, this method can also be repeated multiple times to produce microsale arrays of multicolor pixels for PeLEDs (Figure 9c). In another work, Lin and coworkers developed a dry lift‐off process‐based photolithographic method for patterning MHP films using parylene as an intermediary. Without the aid of orthogonal solvents, single color patterns have been fabricated with pattern resolutions of down to 4 μm. Due to the protection of MHPs by parylene films, standard photolithography processes can be applied multiple times to enable multicolor MHP patterns for PeLEDs (Figure 9d).^[^ ^144^ ^]^ Thermal nanoimprint lithography can be another way of patterning MHPs, which has been used to produce MAPbI_3_ photonic nanostructures for low threshold lasing.^[^ ^145^ ^]^ While all these results are encouraging, further research is needed to achieve large‐scale, high‐resolution patterning of MHPs for PeLED display applications.

Vapor deposition is a well‐established thin‐film preparation technique compatible with mass manufacturing, which has achieved tremendous success in OLED industry. Although it is more expensive and time‐consuming than many solution‐processing techniques, it can afford facile high‐resolution patterning using shadow masks. Indeed, fine metal mask (FMM) patterning is the key technology for realizing the red‐green‐blue side‐by‐side OLED displays. The use of vapor deposition for fabrication of PeLEDs dates back to 2016, when NIR PeLEDs based on MAPbI_3_ were reported with modest performance.^[^ ^146^ ^]^ Following that, PeLEDs fabricated by vapor deposition were reported by many research groups (Figure 9d). ^[^ ^147^, ^148^, ^149^, ^150^ ^]^ However, the device performance of vapor‐deposited PeLEDs reported to date lags far behind those of hybrid‐processed PeLEDs, where MHP layers are prepared via solution processing and CTLs are prepared by either solution processing or vapor deposition. As vapor deposition often forms polycrystalline MHP thin films, it is challenging to realize high exciton binding energies and PLQEs. Patterning vapor‐deposited PeLEDs would not be meaningful until devices with high efficiency are realized.

## 3. Conclusion and Outlook

Remarkable progress has been recorded for PeLEDs, with device efficiencies near the theoretical maxima achieved only 4 years after the first demonstration of room‐temperature electroluminescence. Record‐breaking performance in terms of both efficiency and stability keeps getting refreshed rapidly, giving high hopes of commercialization of PeLEDs in the near future. These tremendous achievements in PeLEDs in a relatively short time are partially attributed to the great experience and knowledge accumulated in decades of research on relevant LED technologies, including OLEDs, QDLEDs, and conventional inorganic LEDs. From material chemistry to device engineering, many well established strategies in those LEDs have been adopted to PeLEDs, although the fundamental differences in chemical and physical properties between MHPs and other types of emitting materials compel specialized solutions to the unique issues and challenges of PeLEDs. In particular, ion migration in PeLEDs is considered as one of the major issues causing low stability of PeLEDs, which is not encountered in other types of LEDs. Can PeLEDs repeat the success of OLEDs to become one of the next‐generation LED technologies for display and lighting applications? We are optimistic and the question is how and when we would get there. Here we outline several research and development directions for PeLEDs in the near term to push them a step closer to commercialization.

1) With the realization of efficient color pure green and red PeLEDs, the development of comparably efficient blue PeLEDs is necessary for applications in full‐color displays. Several strategies have already been reported to obtain blue emitting thin films with high PLQEs, but the challenge remains in engineering efficient charge injection and charge balance. To overcome these problems, modification of the EML, CTL, and device architecture should be investigated. Energy band edge tuning by modifying the surface dipole moment of QDs has been well established and could afford wide tunability ranges of more than 1 eV.^[^ ^151^, ^152^ ^]^ A similar strategy can be used in PeQDs to obtain good band alignment and prevent large charge injection barriers in PeLEDs that lead to poor charge balance. Alternatively, the exploration of new and modification of existing CTLs with suitable energy levels and charge carrier mobility might be necessary to push blue PeLEDs to their theoretical maximum. The realization of efficient and stable blue PeLEDs would also allow for the fabrication of down‐conversion‐type devices for full‐color displays and lighting applications, in which blue PeLEDs serve as blue emission sources as well as excitation sources for optical pump of green and red emissions. Note that exceptional photoluminescence stability has been achieved for green and red MHP emitters with near‐unity PLQEs.

2) Inspired by the advances of QDLEDs, developing inorganic core−shell PeQDs has the potential to be the solution to many of PeLEDs’ ailments. The formation of a core−shell structure could, in principle, suppress ion migration, improve charge injection and transport, passivate traps, and confine excitons within the core using a wide‐bandgap shell with the formation of type‐I heterostructures.^[^ ^153^ ^]^ Due to the ionic nature of MHPs, forming a covalently bonded inorganic semiconducting shell on MHP cores could be synthetically challenging, requiring efforts from the chemistry community. Protecting MHP cores with robust nontoxic inorganic shells could also address Pb toxicity issues even if lead‐based MHPs are used. Once highly luminescent and stable core−shell PeQDs become available, many strategies for the fabrication of QDLEDs could be directly adopted for PeLEDs, as ion migration would no longer be an issue.

3) For lead‐free PeLEDs, although Sn^2+^‐based PeLEDs seem promising, other perovskite‐related metal halides could offer alternative routes to efficient and stable PeLEDs. The synthesis of efficient, perovskite‐related NCs based on nontoxic abundant metals such as Cu^+^ and Sb^3+^ has been reported recently. Although, the device efficiencies, thus far, are far lower than those of Pb^2+^‐based PeLEDs, they have exhibited impressive operational stability due to the high activation energy for halide vacancy formation and ion migration. Moreover, their high formation energies imbue them with improved chemical, environmental, and thermal stability. However, the electronic properties of this class of materials remain under explored and may hold the key to achieving efficient, stable, and lead‐free PeLEDs.

4) MHP‐based CTLs are another promising application worthy of further exploration. The high and thickness‐independent bipolar charge carrier mobility, tunable energy levels, and facile deposition make MHPs promising candidates for high‐efficiency and high‐brightness thin‐film LEDs. The possibility of fabricating all‐MHP PeLEDs is very intriguing, offering good band alignment, balanced charge injection and transport, as well as energy‐level‐graded heterostructures akin to III−V semiconductor‐based LEDs. However, the success of all‐MHP PeLEDs is predicated on finding orthogonal solvents for the different MHP layers or obtaining high PLQE vapor‐deposited MHPs and, more importantly, mitigating ion migration across CTL/EML interfaces.

5) Finally, with the advent of new display industries, such as augmented reality (AR) and virtual reality (VR), the narrow emissions of PeLEDs could be important in offering life‐like experiences to consumers. Currently, micro‐LEDs and micro‐OLEDs, which can afford high pixel density and high brightness, are in use for applications in AR and VR. Future efforts in PeLEDs could also be directed to investigating the possibility of manufacturing micro‐PeLEDs. The first step in this journey could be adopting MHPs to deposition and patterning technologies already available for micro‐LED displays.

## Conflict of Interest

The authors declare no conflict of interest.
